# Relationship between handgrip strength and timed up-and-go test on hospitalization costs in older adults: a population-based study

**DOI:** 10.1186/s12889-025-21489-x

**Published:** 2025-01-23

**Authors:** Kevin Yiqiang Chua, Kelvin Bryan Tan, Rachel Tong, Jon Barrenetxea, Woon-Puay Koh, Cynthia Chen

**Affiliations:** 1 Integrative Sciences and Engineering Programme, NUS Graduate School, National University of Singapore, Singapore, Singapore; 2 Ministry of Health, Singapore, Singapore; 3 Saw Swee Hock School of Public Health, National University of Singapore, Singapore, Singapore; 4 Health Services and Systems Research, Duke-NUS Medical School Singapore, Singapore, Singapore; 5 Healthy Longevity Translational Research Programme, Yong Loo Lin School of Medicine, National University of Singapore, Singapore, Singapore; 6 Singapore Institute for Clinical Sciences, Agency for Science Technology and Research (A*STAR), Singapore, Singapore; 7 Schaeffer Center for Health Policy & Economics, University of Southern California, Los Angeles, CA USA; 8 Department of Non-communicable Disease Epidemiology, London School of Hygiene & Tropical Medicine, London, England, UK

**Keywords:** Grip, Muscle, Strength, Timed-up-and-go, TUG, Physical, Performance, Healthcare, Cost, Expenditure

## Abstract

**Background:**

Weak handgrip strength and slow timed up-and-go (TUG) time are known risk factors for hospitalization among older adults; however, few studies have investigated the relationships between these physical tests and future hospitalization costs.

**Methods:**

We used data from 13,613 participants in the population-based Singapore Chinese Health Study who underwent assessment for handgrip strength and TUG time at a mean age of 74 years. Hospitalization costs for the subsequent year, among those who survived for at least one year thereafter, were ascertained via linkage with administrative healthcare finance data. We analyzed costs using a two-part model that contained a probit regression model in the first part, and a generalized linear regression model with gamma distribution and log link in the second.

**Results:**

Handgrip strength showed a dose-dependent inverse relationship with hospitalization costs (*P*_trend_<0.001). Compared to the strongest quartile, participants in the weakest quartile experienced a 38.2% (95% CI: 18.0-58.5%) increase of US$599 (US$281-US$917) in mean costs. Conversely, TUG time demonstrated a dose-dependent positive association with hospitalization costs (*P*_trend_<0.001). Compared to the fastest quartile, participants in the slowest quartile had a 103.0% (72.1-133.9%) increase of US$1431 (US$1002-US$1859) in mean costs. We then examined combinations of handgrip strength and TUG time. Compared to participants who were both strong and fast, participants who were either weak or slow only had 12.9–48.7% higher mean costs. Meanwhile, participants who were both weak and slow experienced a 99.9% (68.5-131.4%) increase of US$1630 (US$1116-US$2144) in mean costs.

**Conclusions:**

Weak handgrip strength and slow TUG time were independently associated with increased hospitalization costs among older adults.

**Supplementary Information:**

The online version contains supplementary material available at 10.1186/s12889-025-21489-x.

## Background

Globally, US$9 trillion was spent on health in the year 2014, and this expenditure is forecast to increase to US$24 trillion by the year 2040 [1]. A recent systematic review showed that the top 10% of patients accounted for up to 68% of the total costs within a given year [2], providing evidence that the distribution of healthcare spending is skewed with the majority of costs being incurred by a disproportionately small number of patients [2–4]. These patients comprise a very heterogeneous population, and often receive unnecessary or ineffective care while their critical needs remain unmet [2, 4, 5]. The ability to identify this group of individuals would allow for the provision of targeted support and/or preventive interventions that could both improve the quality of care and reduce down-stream costs [4, 5]. This is advantageous for both resource allocation and healthcare decision making, and is therefore of interest to policy makers.

Handgrip strength is a simple, non-invasive indicator of overall skeletal muscle strength that has been shown to be predictive of future bone fractures, functional disability, cognitive impairment, and mortality in older adults [6–12]. Studies have found handgrip strength to be associated with hospital admissions [13–15], complications [16–19], length of stay [20–23], and readmissions [13, 24, 25], but up until now, only one study has investigated its relationship with future hospitalization costs [26]. Similarly, the timed up-and-go (TUG) test [27] is a reliable indicator of physical performance [28, 29] that has been shown to be longitudinally associated with a range of adverse outcomes in older adults, including falls [28–32], functional disability [33], dementia [34], Parkinson’s disease [35], and mortality [12, 36–41]. Several studies have examined its association with hospital admissions [33, 42–45], complications [46], length of stay [47–49], and readmissions [49], but to our best knowledge, none have looked into its relationship with future hospitalization costs.

Therefore, our study aimed to assess whether these physical tests could serve as valuable predictors of hospitalization costs in older adults. Specifically, we examined the prospective associations of handgrip strength and TUG time with hospitalization costs over the subsequent year in a population of community-dwelling older Chinese adults residing in Singapore. As these physical tests have previously been shown to be predictive of short-term mortality [12], and decedents may have had different healthcare needs compared to survivors [50–53], only participants who survived for at least one year following their physical tests were included in this study.

## Methods

### Study population

This study was nested within a prospective, population-based cohort known as the Singapore Chinese Health Study (SCHS) [54]. Between April 1993 and December 1998, the SCHS recruited 63,257 participants (27,959 men and 35,298 women) who were 45–74 (mean: 53) years old. All participants were Chinese who belonged to either of the two major Chinese dialect groups in Singapore, the Hokkien and the Cantonese, who respectively originated from the contiguous provinces of Fujian and Guangdong in the southern part of China. Participants were citizens or permanents residents who resided in government-built housing estates, where 86% of all Singaporeans then resided. After recruitment, consenting survivors were re-contacted for follow-up interviews every five to six years. In this study, we used data collected from 13,613 older adults who had (1) agreed to participate in the third follow-up interviews that were conducted in-person from July 2014 to December 2017, and (2) survived for at least one year after this interview. This study was approved by the Institutional Review Board at the National University of Singapore, and written informed consent was obtained from all study participants.

### Assessment of handgrip strength and timed up-and-go (TUG) time

During the third follow-up interviews, each participant’s handgrip strength was measured by a single trained interviewer in one continuous session, using a digital grip strength dynamometer (Model T.K.K.5401 GRIP D; Takei Scientific Instruments Co., Ltd., Tokyo, Japan) in accordance with standard protocols [12]. Participants clasped the dynamometer with their full force while standing upright with their arms let down naturally. Two trials were performed for each hand, in succession, beginning with the right, and the measurements obtained were recorded to the nearest 0.1 kg. Only the highest value recorded from each hand was retained, and overall handgrip strength was calculated as the mean of the left- and right-hand measurements.

During the same visits, timed up-and-go (TUG) tests [27] were also administered. Participants began by being seated on a chair before being asked to perform the following actions: (1) stand up; (2) walk at their usual pace towards a marker 3 m away; (3) turn around; (4) return to the chair; and (5) sit down again. The time participants took to complete this sequence of actions was measured using a stopwatch and recorded to the nearest second. Two trials were performed in succession, and only the faster time was retained.

### Assessment of hospitalization costs

Hospitalization events and costs in the one year following participants’ physical tests were identified via linkage with administrative healthcare finance data hosted by the Singapore Ministry of Health. This data captured individual-level information of charges and subsidies related to all hospitalizations that occurred in public and private healthcare institutions within the country. Inpatient hospitalization costs were calculated based on total expenditure before government subsidies and insurance claims. These costs were normalized to 2016 Singapore dollars (SGD) using the Consumer Price Index (CPI) for healthcare services [55], and then converted to United States dollars (USD) using the average exchange rate for 2016 (1 USD = 1.3815 SGD) [56].

### Assessment of other covariates

During the third follow-up interviews, a structured interviewer-administered questionnaire was used to collect data on participants’ socioeconomic characteristics, lifestyle factors, and history of physician-diagnosed comorbidities. We screened participants for cognitive impairment, depressive symptoms, anxiety, and functional disability using the Singapore-modified Mini-Mental State Examination (SM-MMSE) [57], the 15-item Geriatric Depression Scale (GDS-15) [58], the Geriatric Anxiety Inventory (GAI) [59], and the Lawton Instrumental Activities of Daily Living (IADL) Scale [60], respectively. Waist circumference was measured 2.5 cm (1 inch) above the navel [61], weight was self-reported, and height was either self-reported or measured at previous follow-up interviews. Mortality in this cohort was identified via linkage with the Singapore Birth and Death Registry through to 31st December 2018.

### Statistical analyses

Of the 13,788 participants who had data on handgrip strength, TUG time, and hospitalization costs, 175 died within one year of their physical tests and were excluded, thus leaving 13,613 participants for this study. For both handgrip strength and TUG time, sex-specific cut-offs were used to define quartile categories for men and women separately [12]. For handgrip strength, the highest (strongest) quartile was used as the referent category; for TUG time, the lowest (fastest) quartile was used. Participants in the weakest sex-specific quartile of handgrip strength were then categorized as ‘weak’, and those in the upper three quartiles were categorized as ‘strong’. Similarly, those with TUG time in the slowest sex-specific quartile were ‘slow’, and those in the lower three quartiles were ‘fast’. Based on the combination of these binary groups, participants were divided into four mutually exclusive categories: ‘strong and fast’ [*N* = 8931 (65.6%)]; ‘weak but fast’ [*N* = 2040 (15.0%)]; ‘strong but slow’ [*N* = 1302 (9.6%)]; and ‘weak and slow’ [*N* = 1341 (9.9%)]. When the physical tests were modeled as continuous variables, participants who had outlying values of handgrip strength or TUG time [values more than 1.5 times the interquartile range (IQR) below Q1 (the lower limit), or 1.5 times the IQR above Q3 (the upper limit)] had their measurements set to the respective lower or upper limit. In this study, the limits for handgrip strength were 1.1 to 40.3 kg, and 83 participants were considered to have outlying values, whereas the limits for TUG time were 3 to 19 s, and 968 participants were considered to have outlying values.

In descriptive analyses, we compared the means [standard deviation (SD)] of continuous variables using one-way analysis of variance (ANOVA), and we compared the proportions of categorical variables using Pearson’s chi-squared test. To estimate the test-retest reliability of participants’ first and second trials of handgrip strength and TUG time, we used two-way mixed-effects models to calculate the individual absolute-agreement intraclass correlation coefficients (ICCs) [62, 63]. As the proportion of participants with no hospitalization costs in the subsequent year was substantial, we utilized two-part models for our regression analyses [64, 65]; these models contained a probit regression model in the first part, and a generalized linear regression model with gamma distribution and log link in the second part [64, 65]. We then used a modified Park test and a Pregibon link test to determine if the specified statistical model was appropriate [65]. Orthogonal polynomial contrasts were used to test for the presence of linear trends. In Model 1, we adjusted for age at physical tests (years) and sex only. In Model 2, we additionally adjusted for socioeconomic characteristics [level of education (no formal education, primary, secondary and above); housing type (1–3 room apartment, 4 room apartment, 5 room or executive apartment, private housing); living arrangement (spouse only, spouse and children, children only, others, alone)], and lifestyle factors [smoking status (never smoker, former smoker, current smoker); body mass index (< 18.5, 18.5–22.9, 23.0–27.4, 27.5 + kg/m^2^); waist circumference (cm)]. In Model 3, we further adjusted for comorbidities [hypertension, coronary artery disease, stroke, diabetes, hypercholesterolemia, gout, cancer, chronic lung disease, bone fractures, arthritis, kidney failure, Parkinson’s disease, cognitive impairment, depression, anxiety] and the presence of functional disability. Finally, in Model 4, the physical tests (handgrip strength or TUG time) were mutually adjusted for (in sex-specific quartiles when modeled categorically, and in kg or sec when modeled continuously). To check for collinearity among the covariates included in our statistical models, we computed their generalized variance inflation factors (GVIFs) (Supplementary Table S1, Additional File 1) [66]. Using a threshold of GVIF > 10 for collinearity [67], we did not find evidence for correlation that was severe enough to warrant corrective measures, as all the covariates in our models had GVIF values of less than 2.00.

Next, we tested for the statistical significance of an interaction between handgrip strength and TUG time by including an additional product term between these two factors into our fully adjusted models; we then obtained a *P*-value for this interaction by performing a likelihood-ratio test between models that did and did not include the aforementioned product term. Additionally, when the physical tests were modeled as continuous variables, we conducted sensitivity analyses that excluded participants with outlying values of handgrip strength or TUG time.

All statistical analyses were conducted using Stata/SE 14.2 software (StataCorp LLC, College Station, TX, USA). All *P*-values presented were two-sided, and *P* < 0.05 was considered statistically significant.

## Results

At the time of their physical tests, participants had a mean (SD) age of 74 (6.2) years, and they ranged from 63 to 97 years old. For handgrip strength, the ICC between participants’ first and second trials were 0.98 (95% CI: 0.98, 0.98) for both left and right hands, indicating a very high degree of test-retest reliability. Moreover, the Pearson’s correlation coefficient between participants’ left- and right-hand measurements was 0.89, indicating good internal reliability. As expected, the men in our study tended to have stronger handgrip strength [mean (SD) of 26.7 (6.31) kg] than the women [mean (SD) of 17.0 (4.22) kg]. As such, we characterized participants based on their sex-specific quartiles of handgrip strength (Table 1). Participants in the weaker quartiles had slower TUG test times, and they were more likely to be older, less educated, and to live in smaller homes either alone or with their children only. Furthermore, these participants were more likely to have lower body mass index, smaller waist circumference, and a history of comorbidities.

**Table 1:** Characteristics of the participants across the sex-specific quartiles of handgrip strength

	Sex-specific quartiles of handgrip strength				*P*-value
	Q4 (Strongest)	Q3	Q2	Q1 (Weakest)	
	(*N* = 3423)	(*N* = 3431)	(*N* = 3378)	(*N* = 3381)	
Mean handgrip strength among men (SD) [kg]	34.66 (3.06)	28.81 (1.17)	24.66 (1.26)	18.59 (3.22)	< 0.001
Mean handgrip strength among women (SD) [kg]	22.36 (2.17)	18.31 (0.81)	15.60 (0.79)	11.64 (1.99)	< 0.001
Mean TUG test time among men (SD) [sec]	9.20 (2.16)	10.31 (4.03)	11.51 (5.42)	14.69 (10.37)	< 0.001
Mean TUG test time among women (SD) [sec]	10.11 (4.14)	11.29 (5.06)	12.78 (7.17)	16.73 (12.45)	< 0.001
Mean age at physical tests (SD) [years]	70.98 (4.72)	72.97 (5.62)	74.92 (6.04)	77.07 (6.57)	< 0.001
Men	1399 (40.9%)	1397 (40.7%)	1398 (41.4%)	1359 (40.2%)	0.80
Level of education					< 0.001
No formal education	461 (13.5%)	540 (15.7%)	641 (19.0%)	757 (22.4%)	
Primary	1396 (40.8%)	1502 (43.8%)	1547 (45.8%)	1604 (47.4%)	
Secondary and above	1566 (45.7%)	1389 (40.5%)	1190 (35.2%)	1020 (30.2%)	
Housing type					< 0.001
1–3 room apartment	991 (29.3%)	1137 (33.5%)	1275 (38.1%)	1467 (43.7%)	
4 room apartment	1335 (39.5%)	1303 (38.3%)	1281 (38.3%)	1207 (35.9%)	
5 room or executive apartment	983 (29.1%)	874 (25.7%)	714 (21.3%)	617 (18.4%)	
Private housing	73 (2.2%)	84 (2.5%)	77 (2.3%)	67 (2.0%)	
Living arrangement					< 0.001
Spouse only	983 (28.7%)	993 (28.9%)	963 (28.5%)	922 (27.3%)	
Spouse and children	1580 (46.2%)	1463 (42.6%)	1292 (38.3%)	1138 (33.7%)	
Children only	524 (15.3%)	613 (17.9%)	682 (20.2%)	837 (24.8%)	
Others	110 (3.2%)	118 (3.4%)	136 (4.0%)	172 (5.1%)	
Alone	226 (6.6%)	244 (7.1%)	304 (9.0%)	312 (9.2%)	
Smoking status					0.08
Never smoker	2680 (78.3%)	2623 (76.5%)	2568 (76.0%)	2546 (75.3%)	
Former smoker	501 (14.6%)	519 (15.1%)	541 (16.0%)	551 (16.3%)	
Current smoker	242 (7.1%)	288 (8.4%)	268 (7.9%)	284 (8.4%)	
Mean BMI (SD) [kg/m^2^]	23.90 (3.51)	23.36 (3.61)	22.92 (3.72)	22.42 (3.87)	< 0.001
Mean waist circumference (SD) [cm]	88.31 (10.34)	87.26 (10.61)	86.57 (11.44)	85.53 (11.42)	< 0.001
Hypertension	1971 (57.6%)	2086 (60.8%)	2141 (63.4%)	2221 (65.7%)	< 0.001
Coronary artery disease	267 (7.8%)	348 (10.1%)	375 (11.1%)	467 (13.8%)	< 0.001
Stroke	89 (2.6%)	148 (4.3%)	174 (5.2%)	239 (7.1%)	< 0.001
Diabetes	549 (16.0%)	719 (21.0%)	788 (23.3%)	897 (26.5%)	< 0.001
Hypercholesterolaemia	1876 (54.8%)	1949 (56.8%)	1998 (59.1%)	1991 (58.9%)	< 0.001
Gout	208 (6.1%)	193 (5.6%)	183 (5.4%)	235 (7.0%)	0.040
Cancer	198 (5.8%)	203 (5.9%)	229 (6.8%)	223 (6.6%)	0.24
Chronic lung disease	19 (0.6%)	22 (0.6%)	35 (1.0%)	45 (1.3%)	0.002
Bone fractures	323 (9.4%)	357 (10.4%)	395 (11.7%)	471 (13.9%)	< 0.001
Arthritis	502 (14.7%)	593 (17.3%)	644 (19.1%)	794 (23.5%)	< 0.001
Kidney failure	16 (0.5%)	37 (1.1%)	29 (0.9%)	68 (2.0%)	< 0.001
Parkinson’s disease	4 (0.1%)	10 (0.3%)	19 (0.6%)	28 (0.8%)	< 0.001
Cognitive impairment	197 (5.8%)	323 (9.4%)	426 (12.6%)	698 (20.6%)	< 0.001
Depression	591 (17.4%)	731 (21.5%)	885 (26.4%)	1170 (34.9%)	< 0.001
Anxiety	239 (7.0%)	309 (9.0%)	359 (10.6%)	561 (16.6%)	< 0.001
Functional disability	978 (28.6%)	1273 (37.1%)	1604 (47.5%)	2103 (62.2%)	< 0.001

Continuous variables were presented as mean (SD), while categorical variables were presented as *N* (%). SD: standard deviation; TUG: timed up-and-go; BMI: body mass index

For the TUG test, the ICC between participants’ first and second trials was also 0.98 (95% CI: 0.98, 0.98), indicating a very high degree of test-retest reliability. Similar to handgrip strength, the men in our study tended to have slightly faster TUG test times [mean (SD) of 11 (6.6) secs] compared to the women [mean (SD) of 13 (8.3) secs]. Hence, we characterized participants based on their sex-specific quartiles of TUG time (Table 2). Participants in the slower quartiles of TUG time had weaker handgrip strength, and they were more likely to be ever-smokers, but were more likely to have higher body mass index and larger waist circumference.

**Table 2:** Characteristics of the participants across the sex-specific quartiles of timed up-and-go (TUG) test time

	Sex-specific quartiles of TUG test time				*P*-value
	Q1 (Fastest)	Q2	Q3	Q4 (Slowest)	
	(*N* = 3997)	(*N* = 3706)	(*N* = 3268)	(*N* = 2642)	
Mean TUG test time among men (SD) [sec]	7.38 (0.80)	9.50 (0.50)	11.82 (0.82)	19.87 (11.93)	< 0.001
Mean TUG test time among women (SD) [sec]	8.05 (0.97)	10.46 (0.51)	12.79 (0.80)	22.65 (13.77)	< 0.001
Mean handgrip strength among men (SD) [kg]	29.67 (5.69)	27.98 (5.61)	25.63 (5.84)	22.04 (5.93)	< 0.001
Mean handgrip strength among women (SD) [kg]	18.84 (3.76)	17.47 (3.91)	16.32 (3.93)	14.26 (3.96)	< 0.001
Mean age at physical tests (SD) [years]	70.44 (4.54)	72.87 (5.26)	75.47 (5.75)	79.01 (6.18)	< 0.001
Men	1407 (35.2%)	1681 (45.4%)	1493 (45.7%)	972 (36.8%)	< 0.001
Level of education					< 0.001
No formal education	336 (8.4%)	544 (14.7%)	669 (20.5%)	850 (32.2%)	
Primary	1492 (37.3%)	1667 (45.0%)	1580 (48.3%)	1310 (49.6%)	
Secondary and above	2169 (54.3%)	1495 (40.3%)	1019 (31.2%)	482 (18.2%)	
Housing type					< 0.001
1–3 room apartment	1153 (29.1%)	1270 (34.7%)	1300 (40.1%)	1147 (43.7%)	
4 room apartment	1485 (37.5%)	1400 (38.2%)	1250 (38.6%)	991 (37.8%)	
5 room or executive apartment	1196 (30.2%)	908 (24.8%)	635 (19.6%)	449 (17.1%)	
Private housing	122 (3.1%)	86 (2.3%)	56 (1.7%)	37 (1.4%)	
Living arrangement					< 0.001
Spouse only	1193 (29.8%)	1133 (30.6%)	883 (27.0%)	652 (24.7%)	
Spouse and children	1851 (46.3%)	1539 (41.5%)	1285 (39.3%)	798 (30.2%)	
Children only	516 (12.9%)	627 (16.9%)	688 (21.1%)	825 (31.2%)	
Others	149 (3.7%)	135 (3.6%)	114 (3.5%)	138 (5.2%)	
Alone	288 (7.2%)	272 (7.3%)	297 (9.1%)	229 (8.7%)	
Smoking status					< 0.001
Never smoker	3330 (83.3%)	2782 (75.1%)	2315 (70.8%)	1990 (75.4%)	
Former smoker	438 (11.0%)	617 (16.6%)	623 (19.1%)	434 (16.4%)	
Current smoker	229 (5.7%)	307 (8.3%)	330 (10.1%)	216 (8.2%)	
Mean BMI (SD) [kg/m^2^]	22.78 (3.31)	23.12 (3.60)	23.35 (3.88)	23.63 (4.21)	< 0.001
Mean waist circumference (SD) [cm]	83.74 (9.85)	86.85 (10.42)	88.10 (11.30)	90.38 (11.74)	< 0.001
Hypertension	2039 (51.0%)	2233 (60.3%)	2197 (67.2%)	1950 (73.8%)	< 0.001
Coronary artery disease	270 (6.8%)	328 (8.9%)	424 (13.0%)	435 (16.5%)	< 0.001
Stroke	69 (1.7%)	132 (3.6%)	155 (4.7%)	294 (11.1%)	< 0.001
Diabetes	612 (15.3%)	730 (19.7%)	798 (24.4%)	813 (30.8%)	< 0.001
Hypercholesterolaemia	2054 (51.4%)	2115 (57.1%)	1996 (61.1%)	1649 (62.4%)	< 0.001
Gout	187 (4.7%)	218 (5.9%)	206 (6.3%)	208 (7.9%)	< 0.001
Cancer	235 (5.9%)	238 (6.4%)	212 (6.5%)	168 (6.4%)	0.69
Chronic lung disease	23 (0.6%)	30 (0.8%)	38 (1.2%)	30 (1.1%)	0.025
Bone fractures	400 (10.0%)	398 (10.7%)	357 (10.9%)	391 (14.8%)	< 0.001
Arthritis	453 (11.3%)	613 (16.5%)	684 (20.9%)	783 (29.6%)	< 0.001
Kidney failure	17 (0.4%)	30 (0.8%)	45 (1.4%)	58 (2.2%)	< 0.001
Parkinson’s disease	2 (0.1%)	9 (0.2%)	16 (0.5%)	34 (1.3%)	< 0.001
Cognitive impairment	227 (5.7%)	320 (8.6%)	444 (13.6%)	653 (24.7%)	< 0.001
Depression	573 (14.5%)	792 (21.5%)	864 (26.6%)	1148 (43.8%)	< 0.001
Anxiety	246 (6.2%)	332 (9.0%)	394 (12.1%)	496 (18.8%)	< 0.001
Functional disability	968 (24.2%)	1348 (36.4%)	1615 (49.4%)	2027 (76.7%)	< 0.001

Continuous variables were presented as mean (SD), while categorical variables were presented as *N* (%). SD: standard deviation; TUG: timed up-and-go; BMI: body mass index

Of the 13,613 participants included in this study, 9483 (69.7%) did not incur any hospitalization costs during the one year after their physical tests. Among the remaining 4130 (30.3%) participants who were hospitalized, the mean costs were US$6343 (SD: US$10,338) and ranged from US$61 to US$152,521. Among participants with non-zero hospitalization costs, a modified Park test returned a coefficient (95% CI) of 2.32 (1.87, 2.77), indicating that the gamma distribution was appropriate. Similarly, a Pregibon link test using a generalized linear model with gamma distribution and log link returned a coefficient for the squared linear predictor that was not statistically significant (*P* = 0.11), indicating that the log link function was appropriate.

In our fully adjusted model, handgrip strength showed a dose-dependent inverse relationship with hospitalization costs (*P*_trend_ < 0.001) (Table 3). When compared to participants in the strongest quartile, those in the weakest quartile experienced a significant 38.2% [95% confidence interval (CI): 18.0%, 58.5%] increase in mean hospitalization costs of US$599 (95% CI: US$281, US$917). When modeled separately as a continuous variable, every 5 kg decrease in handgrip strength was associated with increased mean hospitalization costs of US$241 (95% CI: US$120, US$361). This association remained largely unchanged in sensitivity analyses that excluded participants with outlying values of handgrip strength (Supplementary Table S2, Additional File 1).

**Table 3:** Marginal effects of handgrip strength on the mean increase in hospitalization costs during the subsequent year

				Mean increase in hospitalization costs (95% CI) [2016 USD]			
				Model 1 ^a^	Model 2 ^b^	Model 3 ^c^	Model 4 ^d^
Sex-specific quartiles of handgrip strength		Men [kg]	Women [kg]				
	Q4	(30.9–46.9)	(19.8–33.6)	Ref. ^e^	Ref. ^e^	Ref. ^e^	Ref. ^e^
	Q3	(26.8–30.8)	(17.0–19.7)	$438 ($183, $692)	$455 ($200, $710)	$378 ($108, $647)	$344 ($61, $626)
	Q2	(22.5–26.7)	(14.2–16.9)	$565 ($277, $854)	$623 ($329, $917)	$520 ($212, $829)	$409 ($100, $717)
	Q1	(3.8–22.4)	(2.9–14.1)	$1230 ($921, $1539)	$1256 ($935, $1576)	$858 ($543, $1172)	$599 ($281, $917)
	*P*_trend_			< 0.001	< 0.001	< 0.001	< 0.001
Per 5 kg decrease in handgrip strength				$446 ($338, $553)	$476 ($363, $590)	$340 ($224, $457)	$241 ($120, $361)

Presented as 2016 United States dollars (USD) [95% confidence interval (CI)]. CI: confidence interval; BMI: body mass index; TUG: timed up-and-go

^a^ Model 1: adjusted for age, sex

^b^ Model 2: adjusted for Model 1 and level of education, housing type, living arrangement, smoking status, BMI group, waist circumference

^c^ Model 3: adjusted for Model 2 and hypertension, coronary artery disease, stroke, diabetes, hypercholesterolaemia, gout, cancer, chronic lung disease, bone fractures, arthritis, kidney failure, Parkinson’s disease, cognitive impairment, depression, anxiety, functional disability

^d^ Model 4: adjusted for Model 3 and TUG test time

^e^ Mean (referent) hospitalization costs: $1348 (Model 1); $1327 (Model 2); $1464 (Model 3); $1567 (Model 4)

Meanwhile, TUG time demonstrated a dose-dependent positive association with hospitalization costs (*P*_trend_ < 0.001) (Table 4). Compared to participants in the fastest quartile, those in the slowest quartile had a significant 103.0% (95% CI: 72.1%, 133.9%) increase in mean hospitalization costs of US$1431 (95% CI: US$1002, US$1859). When modeled continuously in separate analyses, each SD (3.5 s) increase in TUG time was associated with increased mean costs of US$465 (95% CI: US$329, US$602). This association remained strong and statistically significant in sensitivity analyses that excluded participants with outlying values of TUG time (Supplementary Table S2, Additional File 1).

**Table 4:** Marginal effects of timed up-and-go (TUG) test time on the mean increase in hospitalization costs during the subsequent year

				Mean increase in hospitalization costs (95% CI) [2016 USD]			
				Model 1 ^a^	Model 2 ^b^	Model 3 ^c^	Model 4 ^d^
Sex-specific quartiles of TUG test time		Men [sec]	Women [sec]				
	Q1	(4–8)	(4–9)	Ref. ^e^	Ref. ^e^	Ref. ^e^	Ref. ^e^
	Q2	(9–10)	(10–11)	$431 ($183, $678)	$434 ($185, $683)	$406 ($147, $665)	$364 ($105, $623)
	Q3	(11–13)	(12–14)	$719 (461, $977)	$734 ($468, $1000)	$571 ($304, $839)	$499 ($227, $771)
	Q4	(14–129)	(15–149)	$2057 ($1663, $2451)	$2038 ($1612, $2464)	$1595 ($1170, $2020)	$1431 ($1002, $1859)
	*P*_trend_			< 0.001	< 0.001	< 0.001	< 0.001
Per SD (3.5 sec) increase in TUG test time				$668 ($554, $782)	$667 ($543, $790)	$536 ($405, $668)	$465 ($329, $602)

Presented as 2016 United States dollars (USD) [95% confidence interval (CI)]. CI: confidence interval; TUG: timed up-and-go; SD: standard deviation; BMI: body mass index

^a^ Model 1: adjusted for age, sex

^b^ Model 2: adjusted for Model 1 and level of education, housing type, living arrangement, smoking status, BMI group, waist circumference

^c^ Model 3: adjusted for Model 2 and hypertension, coronary artery disease, stroke, diabetes, hypercholesterolaemia, gout, cancer, chronic lung disease, bone fractures, arthritis, kidney failure, Parkinson’s disease, cognitive impairment, depression, anxiety, functional disability

^d^ Model 4: adjusted for Model 3 and handgrip strength

^e^ Mean (referent) hospitalization costs: $1222 (Model 1); $1228 (Model 2); $1336 (Model 3); $1389 (Model 4)

We then examined combinations of handgrip strength and TUG time. For this purpose, participants were divided into four mutually exclusive categories (Table 5). Compared to participants who had both strong handgrip strength and fast TUG time, those who had either weak handgrip strength only or slow TUG time only had higher mean hospitalization costs of 12.9% (95% CI: -4.4%, 30.2%) and 48.7% (95% CI: 22.4%, 75.0%), respectively. Meanwhile, participants who had both weak handgrip strength and slow TUG time experienced a significant 99.9% (68.5%, 131.4%) increase in mean hospitalization costs of US$1630 (95% CI: US$1116, US$2144). In a separate model, we assessed handgrip strength and TUG time as continuous variables, and uncovered evidence of an interaction between these physical tests (*P*_interaction_ = 0.001). However, this interaction was not robust, and was no longer statistically significant (*P*_interaction_ = 0.43) in sensitivity analyses that excluded participants with outlying values of handgrip strength or TUG time.

**Table 5:** Marginal effects of the combined handgrip strength and timed up-and-go (TUG) test groups on the mean increase in hospitalization costs in the subsequent year

Mean increase in hospitalization costs (95% CI) [2016 USD] ^a^			
	TUG– Fast (All other quartiles)	TUG– Slow (Slowest quartile)	*P*_interaction_^b^
Grip– Strong (All other quartiles)	Ref. ^c^	$794 ($364, $1223)	0.17
Grip– Weak (Weakest quartile)	$210 (-$72, $492)	$1630 ($1116, $2144)	

Presented as 2016 United States dollars (USD) [95% confidence interval (CI)]. CI: confidence interval; TUG: timed up-and-go; BMI: body mass index

^a^ Adjusted for age, sex, level of education, housing type, living arrangement, smoking status, BMI group, waist circumference, hypertension, coronary artery disease, stroke, diabetes, hypercholesterolaemia, gout, cancer, chronic lung disease, bone fractures, arthritis, kidney failure, Parkinson’s disease, cognitive impairment, depression, anxiety, functional disability

^b^ *P*-value for the likelihood-ratio test between a model that included a product term between weak handgrip strength and slow TUG time against a model that did not

^c^ Mean (referent) hospitalization cost: $1631

## Discussion

In our population of community-dwelling older adults, both weak handgrip strength and slow TUG time demonstrated dose-dependent associations with increased hospitalization costs over the subsequent one year. These associations remained strong and significant even after adjusting for demographics, socioeconomic characteristics, lifestyle factors, comorbidities, and functional disability.

To our best knowledge, only one other study up until now has investigated the relationship between handgrip strength and hospitalization costs. This study by Guerra et al. (2015), which was conducted among Caucasian patients aged 18 years and above who were admitted to a university hospital in Portugal, showed that handgrip strength at the point of admission was associated with subsequent hospitalization costs [26]; when compared to those in the fourth (strongest) quartile, the authors found that patients who were in the first (weakest) or second quartiles of handgrip strength had mean increases in hospitalization costs of 16.6% (95% CI: 1.9%, 31.2%) and 20.0% (95% CI: 6.2%, 33.8%), respectively [26]. To our best knowledge, no prior studies have investigated the relationship between TUG time and subsequent hospitalization costs. Nevertheless, performance on the TUG test is known to be highly correlated with gait speed [68], and a study by Purser et al. (2005), conducted in the United States among acutely ill, hospitalized, older male veterans aged 65 years and above, showed that slower walking speed at the time of admission was associated with increased subsequent hospitalization costs [69]. Our results concurred with the aforementioned studies, but extended their findings to show that even when conducted among community-dwelling older adults who were relatively well at the time, these physical tests were still strongly associated with hospitalization costs over the subsequent one year.

Our finding that handgrip strength and TUG time were associated with subsequent hospitalization costs was not unexpected. These physical tests have been shown in previous studies to be associated with various aspects of hospitalization, including admissions [13–15, 33, 42–45], complications [16–19, 46], length of stay [20–23, 47–49], and readmissions [13, 24, 25, 49]. Furthermore, handgrip strength and TUG time are often used as indicators of low muscle strength and poor physical performance, respectively, in the diagnosis of frailty [70, 71] and sarcopenia [72, 73] among older adults, and both of these syndromes have been shown to be associated with higher hospitalization costs [74–78].

Our findings are relevant in the context of the World Health Organization’s concept of healthy ageing, which is defined as the process of developing and maintaining functional ability that enables well-being in older age [79]. Functional ability is determined by (1) the environment in which a person lives, and by (2) their intrinsic capacity, which is comprised of all the mental and physical capacities that they can draw upon [79]. The handgrip strength and TUG tests, as indicators of muscle strength and physical performance, respectively, could provide insight into a person’s intrinsic capacity, and allow for the provision of more person-centered and integrated models of care. For example, incorporating grip strength measurement into routine assessments could help screen patients at risk of poor healthcare outcomes who might require more resources during inpatient admission [80]. Furthermore, it could also facilitate fast-tracked admission and discharge for patients with normal grip strength, thereby reducing their length of stay [80]. Moreover, this indicator could be integrated into predictive models to identify patients at high risk of future hospital admissions, allowing for targeted interventions to reduce the likelihood of readmissions [81]. The findings of our study show how such physical tests may be used for screening, and reinforce the health economic significance of detecting age-related syndromes and declines in intrinsic capacity, instead of just the presence and absence of chronic diseases. Such changes could allow health systems to better detect and target geriatric syndromes, and therefore promote healthy ageing within a population.

The strengths of our study included its prospective design, use of community-dwelling participants, wide variation in handgrip strengths and TUG times, and complete capture of hospitalization costs through linkage with nationwide administrative healthcare finance data. Furthermore, linkage with the Singapore Birth and Death registry also enabled the complete ascertainment of deaths, and allowed us to only include participants who had survived for at least one year following their physical tests. However, several limitations should be noted. First, the protocols for the measurement of handgrip strength are known to vary considerably across different clinical and epidemiological studies [82]. As a result, our findings might not be directly comparable to those from other studies that had utilized different protocols for the measurement of handgrip strength. Nevertheless, in order to minimize this possibility, we adhered to well-established protocols for both the measurement and computation of participants’ handgrip strength. In our study, handgrip strength measurements were taken with a spring-type (Smedley) dynamometer. Although hydraulic-type (Jamar) dynamometers are generally accepted as the gold standard [82], spring-type dynamometers are widely used around the world [83], and are the type of dynamometers that are used most often in Asia [72]. Studies have found excellent correlations between both types of dynamometers [84], and have shown that measurements from spring-type dynamometers are both reliable and valid [83, 85]. However, despite this, readers should note that the data generated from spring- and hydraulic-type dynamometers may not be directly comparable [72, 83–86]. Regardless, our study obtained handgrip strength measurements from participants in a standing position; this is the most common approach, and the standard positioning recommended by the Asian Working Group for Sarcopenia 2019 when spring-type dynamometers are employed [72]. For the computation of handgrip strength, only the highest value recorded from each hand was retained, and participants’ overall handgrip strength was calculated as the mean of the left- and right-hand measurements. These calculations adhered to the methods utilized by the Prospective Urban and Rural Epidemiological (PURE) study– a large longitudinal population study conducted across 17 countries [87]. Second, as we did not perform the relevant assessment at the third follow-up interviews, our study was not able to investigate the association between hospitalization costs and gait speed. Both the gait speed and TUG tests are often used to assess physical performance in older adults [68, 72, 73, 88, 89], but gait speed is more commonly used in the diagnosis of frailty [70] and sarcopenia [72, 73]. Compared to gait speed, the TUG test involves additional complex functional movements such as standing, turning, and sitting; however, whether or not these elements improve its ability to predict adverse outcomes is a subject of debate [68, 88, 89].

Third, in our study, the data on participants’ comorbidities at the third follow-up interviews were self-reported. Participants’ self-reported comorbidities might not have been entirely accurate, and may not have necessarily aligned with those in their medical records. Moreover, there was a possibility that certain conditions or diseases were underdiagnosed in our study population. Nonetheless, we had previously conducted a validation study in this cohort that involved 1,651 participants who had self-reported a history of physician-diagnosed diabetes mellitus at the baseline interviews [90, 91]. In this validation study, cases of diabetes mellitus were ascertained through linkage with hospital records from a nationwide hospital-based discharge summary database. Additionally, if a participant did not have diabetes-related hospitalization records, they were contacted by telephone to answer a supplementary questionnaire about symptoms, diagnostic tests, and hypoglycemic therapy. On the basis of these criteria, a positive predictive value of 98.8% was observed [90, 91]. Moreover, in our present study, self-reported diabetes mellitus at the third follow-up interviews was associated with a significant increase in mean hospitalization costs of US$396 (95% CI: US$125, US$667) in the subsequent year, as was expected. Other comorbidities, such as hypertension, coronary artery disease, cancer, bone fractures, and kidney failure were also similarly associated with significantly increased mean hospitalization costs (Supplementary Table S3, Additional File 1). Fourth, we acknowledge that the possibility of residual confounding cannot be eliminated. In our study, residual confounding could have arisen from inadequate adjustments of unmeasured confounders, such as the duration and severity of comorbidities. Finally, it remains unclear if our findings are generalizable to older adults from other populations, especially to those who are not of Chinese ethnicity. Further studies in other populations will be required to confirm both the validity and generalizability of our results.

## Conclusions

Our study showed that weak handgrip strength and slow TUG time were independently associated with increased hospitalization costs among community-dwelling older adults. The dose-dependent associations between performance on these physical tests and hospitalization costs lend support to their potential as simple and valuable predictors of future hospitalization costs.

## Electronic supplementary material

Below is the link to the electronic supplementary material.

Additional File 1: Supplementary Tables S1 to S3

## Data Availability

The datasets generated and/or analyzed during the current study are not publicly available due to data privacy laws, but are available from the corresponding author on reasonable request.

## Abbreviations

ANOVA: Analysis of variance
BMI: Body mass index
CI: Confidence interval
CPI: Consumer price index
GAI: Geriatric anxiety inventory
GDS-15: 15-item geriatric depression scale
GVIF: Generalized variance inflation factor
IADL: Instrumental activities of daily living
ICC: Intraclass correlation coefficient
IQR: Interquartile range
PURE: Prospective Urban and Rural Epidemiological
SCHS: Singapore Chinese Health Study
SD: Standard deviation
SGD: Singapore dollars
SM-MMSE: Singapore-modified mini-mental state examination
TUG: Timed up-and-go
USD: United States dollars

## References

[1] Global Burden of Disease Health Financing Collaborator Network. Future and potential spending on health 2015-40: development assistance for health, and government, prepaid private, and out-of-pocket health spending in 184 countries. Lancet. 2017;389(10083):2005–30. 10.1016/S0140-6736(17)30873-5 28433260 10.1016/S0140-6736(17)30873-5 PMC5440765

[2] Wammes JJG, van der Wees PJ, Tanke MAC, Westert GP, Jeurissen PPT. Systematic review of high-cost patients’ characteristics and healthcare utilisation. BMJ Open. 2018;8(9):e023113. 10.1136/bmjopen-2018-023113 30196269 10.1136/bmjopen-2018-023113 PMC6129088

[3] Ng SHX, Rahman N, Ang IYH, Sridharan S, Ramachandran S, Wang DD, et al. Characterising and predicting persistent high-cost utilisers in healthcare: a retrospective cohort study in Singapore. BMJ Open. 2020;10(1):e031622. 10.1136/bmjopen-2019-031622 31911514 10.1136/bmjopen-2019-031622 PMC6955475

[4] Bodenheimer T, Fernandez A. High and rising health care costs. Part 4: can costs be controlled while preserving quality? Ann Intern Med. 2005;143(1):26–31. 10.7326/0003-4819-143-1-200507050-00007 15998752 10.7326/0003-4819-143-1-200507050-00007

[5] Blumenthal D, Chernof B, Fulmer T, Lumpkin J, Selberg J. Caring for High-Need, high-cost patients - an Urgent Priority. N Engl J Med. 2016;375(10):909–11. 10.1056/NEJMp1608511 27602661 10.1056/NEJMp1608511

[6] Bohannon RW. Grip strength: an indispensable biomarker for older adults. Clin Interv Aging. 2019;14:1681–91. 10.2147/CIA.S194543 31631989 10.2147/CIA.S194543 PMC6778477

[7] Carson RG. Get a grip: individual variations in grip strength are a marker of brain health. Neurobiol Aging. 2018;71:189–222. 10.1016/j.neurobiolaging.2018.07.023 30172220 10.1016/j.neurobiolaging.2018.07.023

[8] Rijk JM, Roos PR, Deckx L, van den Akker M, Buntinx F. Prognostic value of handgrip strength in people aged 60 years and older: a systematic review and meta-analysis. Geriatr Gerontol Int. 2016;16(1):5–20. 10.1111/ggi.12508 26016893 10.1111/ggi.12508

[9] Fritz NE, McCarthy CJ, Adamo DE. Handgrip strength as a means of monitoring progression of cognitive decline - A scoping review. Ageing Res Rev. 2017;35:112–23. 10.1016/j.arr.2017.01.004 28189666 10.1016/j.arr.2017.01.004

[10] McGrath R, Johnson N, Klawitter L, Mahoney S, Trautman K, Carlson C, et al. What are the association patterns between handgrip strength and adverse health conditions? A topical review. SAGE Open Med. 2020;8:2050312120910358. 10.1177/2050312120910358 32166029 10.1177/2050312120910358 PMC7052448

[11] Norman K, Stobaus N, Gonzalez MC, Schulzke JD, Pirlich M. Hand grip strength: outcome predictor and marker of nutritional status. Clin Nutr. 2011;30(2):135–42. 10.1016/j.clnu.2010.09.010 21035927 10.1016/j.clnu.2010.09.010

[12] Chua KY, Lim WS, Lin X, Yuan JM, Koh WP. Handgrip strength and timed Up-and-Go (TUG) test are predictors of short-term mortality among Elderly in a Population-based cohort in Singapore. J Nutr Health Aging. 2020;24(4):371–8. 10.1007/s12603-020-1337-032242204 10.1007/s12603-020-1337-0

[13] Simmonds SJ, Syddall HE, Westbury LD, Dodds RM, Cooper C, Aihie Sayer A. Grip strength among community-dwelling older people predicts hospital admission during the following decade. Age Ageing. 2015;44(6):954–9. 10.1093/ageing/afv146 26504117 10.1093/ageing/afv146 PMC6485460

[14] Cawthon PM, Fox KM, Gandra SR, Delmonico MJ, Chiou CF, Anthony MS, et al. Do muscle mass, muscle density, strength, and physical function similarly influence risk of hospitalization in older adults? J Am Geriatr Soc. 2009;57(8):1411–9. 10.1111/j.1532-5415.2009.02366.x 19682143 10.1111/j.1532-5415.2009.02366.x PMC3269169

[15] Hamasaki H, Kawashima Y, Katsuyama H, Sako A, Goto A, Yanai H. Association of handgrip strength with hospitalization, cardiovascular events, and mortality in Japanese patients with type 2 diabetes. Sci Rep. 2017;7(1):7041. 10.1038/s41598-017-07438-8 28765572 10.1038/s41598-017-07438-8 PMC5539205

[16] Alvares-da-Silva MR, Reverbel da Silveira T. Comparison between handgrip strength, subjective global assessment, and prognostic nutritional index in assessing malnutrition and predicting clinical outcome in cirrhotic outpatients. Nutrition. 2005;21(2):113–7. 10.1016/j.nut.2004.02.002 15723736 10.1016/j.nut.2004.02.002

[17] Karlsson E, Egenvall M, Farahnak P, Bergenmar M, Nygren-Bonnier M, Franzen E, et al. Better preoperative physical performance reduces the odds of complication severity and discharge to care facility after abdominal cancer resection in people over the age of 70 - a prospective cohort study. Eur J Surg Oncol. 2018;44(11):1760–7. 10.1016/j.ejso.2018.08.011 30201418 10.1016/j.ejso.2018.08.011

[18] Perry IS, Pinto LC, da Silva TK, Vieira SRR, Souza GC. Handgrip strength in preoperative elective cardiac surgery patients and Association with Body Composition and Surgical Risk. Nutr Clin Pract. 2019;34(5):760–6. 10.1002/ncp.10267 30864228 10.1002/ncp.10267

[19] Gonzalez EDL, Mendivil LLL, Garza DPS, Hermosillo HG, Chavez JHM, Corona RP. Low handgrip strength is associated with a higher incidence of pressure ulcers in hip fractured patients. Acta Orthop Belg. 2018;84(3):284–91. 30840570

[20] Savino E, Sioulis F, Guerra G, Cavalieri M, Zuliani G, Guralnik JM, et al. Potential prognostic value of Handgrip Strength in older hospitalized patients. J Frailty Aging. 2012;1(1):32–8. 10.14283/jfa.2012.6 27092935 10.14283/jfa.2012.6

[21] Vecchiarino P, Bohannon RW, Ferullo J, Maljanian R. Short-term outcomes and their predictors for patients hospitalized with community-acquired pneumonia. Heart Lung. 2004;33(5):301–7. 10.1016/j.hrtlng.2004.03.007 15454909 10.1016/j.hrtlng.2004.03.007

[22] Shyam Kumar AJ, Beresford-Cleary N, Kumar P, Barai A, Vasukutty N, Yasin S, et al. Preoperative grip strength measurement and duration of hospital stay in patients undergoing total hip and knee arthroplasty. Eur J Orthop Surg Traumatol. 2013;23(5):553–6. 10.1007/s00590-012-1029-5 23412162 10.1007/s00590-012-1029-5

[23] Yost G, Bhat G. Relationship between handgrip strength and length of stay for left ventricular assist device implantation. Nutr Clin Pract. 2017;32(1):98–102. 10.1177/0884533616665926 27600398 10.1177/0884533616665926

[24] Andreasen J, Aadahl M, Sorensen EE, Eriksen HH, Lund H, Overvad K. Associations and predictions of readmission or death in acutely admitted older medical patients using self-reported frailty and functional measures. A Danish cohort study. Arch Gerontol Geriatr. 2018;76:65–72. 10.1016/j.archger.2018.01.013 29462759 10.1016/j.archger.2018.01.013

[25] Allard JP, Keller H, Teterina A, Jeejeebhoy KN, Laporte M, Duerksen DR, et al. Lower handgrip strength at discharge from acute care hospitals is associated with 30-day readmission: a prospective cohort study. Clin Nutr. 2016;35(6):1535–42. 10.1016/j.clnu.2016.04.008 27155939 10.1016/j.clnu.2016.04.008

[26] Guerra RS, Amaral TF, Sousa AS, Pichel F, Restivo MT, Ferreira S, et al. Handgrip strength measurement as a predictor of hospitalization costs. Eur J Clin Nutr. 2015;69(2):187–92. 10.1038/ejcn.2014.242 25369830 10.1038/ejcn.2014.242

[27] Podsiadlo D, Richardson S. The timed up & go: a test of basic functional mobility for frail elderly persons. J Am Geriatr Soc. 1991;39(2):142–8. 10.1111/j.1532-5415.1991.tb01616.x 1991946 10.1111/j.1532-5415.1991.tb01616.x

[28] Christopher A, Kraft E, Olenick H, Kiesling R, Doty A. The reliability and validity of the timed up and go as a clinical tool in individuals with and without disabilities across a lifespan: a systematic review. Disabil Rehabil. 2021;43(13):1799–813. 10.1080/09638288.2019.1682066 31656104 10.1080/09638288.2019.1682066

[29] Rydwik E, Bergland A, Forsén L, Frändin K. Psychometric properties of timed up and go in Elderly people: a systematic review. Phys Occup Ther Geriatr. 2011;29(2):102–25. 10.3109/02703181.2011.564725

[30] Beauchet O, Fantino B, Allali G, Muir SW, Montero-Odasso M, Annweiler C. Timed up and go test and risk of falls in older adults: a systematic review. J Nutr Health Aging. 2011;15(10):933–8. 10.1007/s12603-011-0062-022159785 10.1007/s12603-011-0062-0

[31] Barry E, Galvin R, Keogh C, Horgan F, Fahey T. Is the timed up and go test a useful predictor of risk of falls in community dwelling older adults: a systematic review and meta-analysis. BMC Geriatr. 2014;14:14. 10.1186/1471-2318-14-14 24484314 10.1186/1471-2318-14-14 PMC3924230

[32] Schoene D, Wu SM, Mikolaizak AS, Menant JC, Smith ST, Delbaere K, et al. Discriminative ability and predictive validity of the timed up and go test in identifying older people who fall: systematic review and meta-analysis. J Am Geriatr Soc. 2013;61(2):202–8. 10.1111/jgs.12106 23350947 10.1111/jgs.12106

[33] Gobbens RJ, van Assen MA. Frailty and its prediction of disability and health care utilization: the added value of interviews and physical measures following a self-report questionnaire. Arch Gerontol Geriatr. 2012;55(2):369–79. 10.1016/j.archger.2012.04.008 22595766 10.1016/j.archger.2012.04.008

[34] de Oliveira Silva F, Ferreira JV, Placido J, Chagas D, Praxedes J, Guimaraes C, et al. Stages of mild cognitive impairment and Alzheimer’s disease can be differentiated by declines in timed up and go test: a systematic review and meta-analysis. Arch Gerontol Geriatr. 2019;85:103941. 10.1016/j.archger.2019.103941 31476630 10.1016/j.archger.2019.103941

[35] Yoo JE, Jang W, Shin DW, Jeong SM, Jung HW, Youn J, et al. Timed up and go test and the risk of Parkinson’s Disease: a Nation-wide retrospective cohort study. Mov Disord. 2020;35(7):1263–7. 10.1002/mds.28055 32293759 10.1002/mds.28055

[36] Bergland A, Jorgensen L, Emaus N, Strand BH. Mobility as a predictor of all-cause mortality in older men and women: 11.8 year follow-up in the Tromso study. BMC Health Serv Res. 2017;17(1):22. 10.1186/s12913-016-1950-0 28068995 10.1186/s12913-016-1950-0 PMC5223479

[37] De Buyser S, Petrovic M, Taes Y, Toye K, Kaufman JM, Goemaere S, et al. Three year functional changes and long-term mortality hazard in community-dwelling older men. Eur J Intern Med. 2016;35:66–72. 10.1016/j.ejim.2016.06.006 27378504 10.1016/j.ejim.2016.06.006

[38] De Buyser SL, Petrovic M, Taes YE, Toye KR, Kaufman JM, Goemaere S. Physical function measurements predict mortality in ambulatory older men. Eur J Clin Invest. 2013;43(4):379–86. 10.1111/eci.12056 23398295 10.1111/eci.12056

[39] Idland G, Engedal K, Bergland A. Physical performance and 13.5-year mortality in elderly women. Scand J Public Health. 2013;41(1):102–8. 10.1177/1403494812466460 23178925 10.1177/1403494812466460

[40] Sim M, Prince RL, Scott D, Daly RM, Duque G, Inderjeeth CA, et al. Sarcopenia definitions and their associations with Mortality in older Australian women. J Am Med Dir Assoc. 2019;20(1):76–e822. 10.1016/j.jamda.2018.10.016 30527277 10.1016/j.jamda.2018.10.016

[41] Tice JA, Kanaya A, Hue T, Rubin S, Buist DS, Lacroix A, et al. Risk factors for mortality in middle-aged women. Arch Intern Med. 2006;166(22):2469–77. 10.1001/archinte.166.22.2469 17159012 10.1001/archinte.166.22.2469

[42] Batko-Szwaczka A, Wilczynski K, Hornik B, Janusz-Jenczen M, Wlodarczyk I, Wnuk B, et al. Predicting adverse outcomes in healthy Aging Community-Dwelling early-old adults with the timed up and go test. Clin Interv Aging. 2020;15:1263–70. 10.2147/CIA.S256312 32801674 10.2147/CIA.S256312 PMC7402859

[43] Chun SH, Cho B, Yang HK, Ahn E, Han MK, Oh B, et al. Performance on physical function tests and the risk of fractures and admissions: findings from a national health screening of 557,648 community-dwelling older adults. Arch Gerontol Geriatr. 2017;68:174–80. 10.1016/j.archger.2016.10.008 27810666 10.1016/j.archger.2016.10.008

[44] Hassan A, Wu SS, Schmidt P, Dai Y, Simuni T, Giladi N, et al. High rates and the risk factors for emergency room visits and hospitalization in Parkinson’s disease. Parkinsonism Relat Disord. 2013;19(11):949–54. 10.1016/j.parkreldis.2013.06.006 23835430 10.1016/j.parkreldis.2013.06.006

[45] Shahgholi L, De Jesus S, Wu SS, Pei Q, Hassan A, Armstrong MJ, et al. Hospitalization and rehospitalization in Parkinson disease patients: data from the National Parkinson Foundation Centers of Excellence. PLoS ONE. 2017;12(7):e0180425. 10.1371/journal.pone.0180425 28683150 10.1371/journal.pone.0180425 PMC5500337

[46] Huisman MG, van Leeuwen BL, Ugolini G, Montroni I, Spiliotis J, Stabilini C, et al. Timed up & go: a screening tool for predicting 30-day morbidity in onco-geriatric surgical patients? A multicenter cohort study. PLoS ONE. 2014;9(1):e86863. 10.1371/journal.pone.0086863 24475186 10.1371/journal.pone.0086863 PMC3901725

[47] Petis SM, Howard JL, Lanting BA, Somerville LE, Vasarhelyi EM. Perioperative predictors of length of stay after total hip arthroplasty. J Arthroplasty. 2016;31(7):1427–30. 10.1016/j.arth.2016.01.005 26869060 10.1016/j.arth.2016.01.005

[48] Basic D, Khoo A. Admission variables predicting short lengths of stay of acutely unwell older patients: relevance to emergency and medical short-stay units. Aust Health Rev. 2009;33(3):502–12. 10.1071/ah090502 20128769 10.1071/ah090502

[49] Wong RY, Miller WC. Adverse outcomes following hospitalization in acutely ill older patients. BMC Geriatr. 2008;8:10. 10.1186/1471-2318-8-10 18479512 10.1186/1471-2318-8-10 PMC2391142

[50] Alipour V, Pourreza A, Kosheshi M, Heydari H, Emamgholipour Sefiddashti S. Hospital expenditure at the end-of-Life: a time-to-death Approach. Int J Health Policy Manag. 2022;11(2):138–44. 10.34172/ijhpm.2020.88 32610810 10.34172/ijhpm.2020.88 PMC9278609

[51] Duncan I, Ahmed T, Dove H, Maxwell TL. Medicare Cost at end of life. Am J Hosp Palliat Care. 2019;36(8):705–10. 10.1177/1049909119836204 30884954 10.1177/1049909119836204 PMC6610551

[52] Luta X, Diernberger K, Bowden J, Droney J, Howdon D, Schmidlin K, et al. Healthcare trajectories and costs in the last year of life: a retrospective primary care and hospital analysis. BMJ Support Palliat Care. 2020. 10.1136/bmjspcare-2020-002630 33268473 10.1136/bmjspcare-2020-002630

[53] Mallow PJ, Browne F, Shemisa K. The high cost of death after Acute Myocardial infarctions:results from a National US Hospital Database. Clinicoecon Outcomes Res. 2023;15:63–8. 10.2147/CEOR.S397220 10.2147/CEOR.S397220 PMC9899015 36747496

[54] Hankin JH, Stram DO, Arakawa K, Park S, Low SH, Lee HP, et al. Singapore Chinese Health Study: development, validation, and calibration of the quantitative food frequency questionnaire. Nutr Cancer. 2001;39(2):187–95. 10.1207/S15327914nc392_5 11759279 10.1207/S15327914nc392_5

[55] Ministry of Health Singapore. Consumer Price Indices (CPI) & Household Healthcare Expenditure. https://www.moh.gov.sg/resources-statistics/singapore-health-facts/consumer-price-indices-(cpi)-household-healthcare-expenditure (2018). Accessed 10 April 2023 2023.

[56] Department of Statistics Singapore. Exchange Rates (Average For The Year). https://tablebuilder.singstat.gov.sg/table/TS/M700851 (2023). Accessed 10 April 2023 2023.

[57] Feng L, Chong MS, Lim WS, Ng TP. The modified Mini-mental State Examination test: normative data for Singapore Chinese older adults and its performance in detecting early cognitive impairment. Singap Med J. 2012;53(7):458–62. 22815014

[58] Sheikh JI, Yesavage JA. Geriatric Depression Scale (GDS): recent evidence and development of a shorter version. Clin Gerontologist: J Aging Mental Health. 1986;5(1–2):165–73. 10.1300/J018v05n01_09

[59] Pachana NA, Byrne GJ, Siddle H, Koloski N, Harley E, Arnold E. Development and validation of the geriatric anxiety inventory. Int Psychogeriatr. 2007;19(1):103–14. 10.1017/S1041610206003504 16805925 10.1017/S1041610206003504

[60] Lawton MP, Brody EM. Assessment of older people: self-maintaining and instrumental activities of daily living. Gerontologist. 1969;9(3):179–86. 5349366

[61] Guerra RS, Amaral TF, Marques EA, Mota J, Restivo MT. Anatomical location for waist circumference measurement in older adults: a preliminary study. Nutr Hosp. 2012;27(5):1554–61. 10.3305/nh.2012.27.5.5922 23478705 10.3305/nh.2012.27.5.5922

[62] Koo TK, Li MY. A Guideline of selecting and reporting Intraclass correlation coefficients for Reliability Research. J Chiropr Med. 2016;15(2):155–63. 10.1016/j.jcm.2016.02.012 27330520 10.1016/j.jcm.2016.02.012 PMC4913118

[63] Qin S, Nelson L, McLeod L, Eremenco S, Coons SJ. Assessing test-retest reliability of patient-reported outcome measures using intraclass correlation coefficients: recommendations for selecting and documenting the analytical formula. Qual Life Res. 2019;28(4):1029–33. 10.1007/s11136-018-2076-0 30547346 10.1007/s11136-018-2076-0 PMC6439259

[64] Chen S, Zhou XA. Modeling and analysis of cost data. In: Sobolev B, Gatsonis C, editors. Methods in Health Services Research. New York, NY: Springer US; 2018. pp. 1–32.

[65] Zhou J, Williams C, Keng MJ, Wu R, Mihaylova B. Estimating costs Associated with Disease Model States using generalized Linear models: a Tutorial. PharmacoEconomics. 2024;42(3):261–73. 10.1007/s40273-023-01319-x 37948040 10.1007/s40273-023-01319-x PMC11424740

[66] Fox J, Monette G. Generalized Collinearity Diagnostics. J Am Stat Assoc. 1992;87(417):178–83. 10.2307/2290467

[67] Dormann CF, Elith J, Bacher S, Buchmann C, Carl G, Carré G, et al. Collinearity: a review of methods to deal with it and a simulation study evaluating their performance. Ecography. 2012;36(1):27–46. 10.1111/j.1600-0587.2012.07348.x

[68] Viccaro LJ, Perera S, Studenski SA. Is timed up and go better than gait speed in predicting health, function, and falls in older adults? J Am Geriatr Soc. 2011;59(5):887–92. 10.1111/j.1532-5415.2011.03336.x 21410448 10.1111/j.1532-5415.2011.03336.x PMC3522463

[69] Purser JL, Weinberger M, Cohen HJ, Pieper CF, Morey MC, Li T, et al. Walking speed predicts health status and hospital costs for frail elderly male veterans. J Rehabil Res Dev. 2005;42(4):535–46. 10.1682/jrrd.2004.07.0087 16320148 10.1682/jrrd.2004.07.0087

[70] Fried LP, Tangen CM, Walston J, Newman AB, Hirsch C, Gottdiener J, et al. Frailty in older adults: evidence for a phenotype. J Gerontol Biol Sci Med Sci. 2001;56(3):M146–56. 10.1093/gerona/56.3.m146 10.1093/gerona/56.3.m146 11253156

[71] Savva GM, Donoghue OA, Horgan F, O’Regan C, Cronin H, Kenny RA. Using timed up-and-go to identify frail members of the older population. J Gerontol Biol Sci Med Sci. 2013;68(4):441–6. 10.1093/gerona/gls190 10.1093/gerona/gls190 22987796

[72] Chen LK, Woo J, Assantachai P, Auyeung TW, Chou MY, Iijima K et al. Asian Working Group for Sarcopenia: 2019 Consensus Update on Sarcopenia Diagnosis and Treatment. J Am Med Dir Assoc. 2020;21(3):300-7 e2. 10.1016/j.jamda.2019.12.012 10.1016/j.jamda.2019.12.012 32033882

[73] Cruz-Jentoft AJ, Bahat G, Bauer J, Boirie Y, Bruyere O, Cederholm T, et al. Sarcopenia: revised European consensus on definition and diagnosis. Age Ageing. 2019;48(1):16–31. 10.1093/ageing/afy169 30312372 10.1093/ageing/afy169 PMC6322506

[74] Norman K, Otten L. Financial impact of Sarcopenia or low muscle mass - A short review. Clin Nutr. 2019;38(4):1489–95. 10.1016/j.clnu.2018.09.026 30316536 10.1016/j.clnu.2018.09.026

[75] Janssen I, Shepard DS, Katzmarzyk PT, Roubenoff R. The healthcare costs of Sarcopenia in the United States. J Am Geriatr Soc. 2004;52(1):80–5. 10.1111/j.1532-5415.2004.52014.x 14687319 10.1111/j.1532-5415.2004.52014.x

[76] Chi J, Chen F, Zhang J, Niu X, Tao H, Ruan H, et al. Impacts of frailty on health care costs among community-dwelling older adults: a meta-analysis of cohort studies. Arch Gerontol Geriatr. 2021;94:104344. 10.1016/j.archger.2021.104344 33516075 10.1016/j.archger.2021.104344

[77] Kojima G. Increased healthcare costs associated with frailty among community-dwelling older people: a systematic review and meta-analysis. Arch Gerontol Geriatr. 2019;84:103898. 10.1016/j.archger.2019.06.003 31228673 10.1016/j.archger.2019.06.003

[78] Hoogendijk EO, Afilalo J, Ensrud KE, Kowal P, Onder G, Fried LP. Frailty: implications for clinical practice and public health. Lancet. 2019;394(10206):1365–75. 10.1016/S0140-6736(19)31786-6 31609228 10.1016/S0140-6736(19)31786-6

[79] Beard JR, Officer A, de Carvalho IA, Sadana R, Pot AM, Michel JP, et al. The World report on ageing and health: a policy framework for healthy ageing. Lancet. 2016;387(10033):2145–54. 10.1016/S0140-6736(15)00516-4 26520231 10.1016/S0140-6736(15)00516-4 PMC4848186

[80] Ibrahim K, May CR, Patel HP, Baxter M, Sayer AA, Roberts HC. Implementation of grip strength measurement in medicine for older people wards as part of routine admission assessment: identifying facilitators and barriers using a theory-led intervention. BMC Geriatr. 2018;18(1):79. 10.1186/s12877-018-0768-5 29566673 10.1186/s12877-018-0768-5 PMC5865333

[81] Ta WA, Lee KH, Wong SF, Chow WL, Kumar PV, Choong ZC et al. Development and implementation of nationwide predictive model for admission prevention: System architecture & machine learning. 2018 IEEE EMBS International Conference on Biomedical & Health Informatics (BHI). 2018:303-6. 10.1109/bhi.2018.8333429

[82] Roberts HC, Denison HJ, Martin HJ, Patel HP, Syddall H, Cooper C, et al. A review of the measurement of grip strength in clinical and epidemiological studies: towards a standardised approach. Age Ageing. 2011;40(4):423–9. 10.1093/ageing/afr051 21624928 10.1093/ageing/afr051

[83] Savas S, Kilavuz A, Kayhan Kocak FO, Cavdar S. Comparison of grip strength measurements by widely used three dynamometers in outpatients aged 60 years and over. J Clin Med. 2023;12(13). 10.3390/jcm12134260 10.3390/jcm12134260 PMC10342845 37445293

[84] Kim M, Shinkai S. Prevalence of muscle weakness based on different diagnostic criteria in community-dwelling older adults: a comparison of grip strength dynamometers. Geriatr Gerontol Int. 2017;17(11):2089–95. 10.1111/ggi.13027 28517036 10.1111/ggi.13027

[85] Huang L, Liu Y, Lin T, Hou L, Song Q, Ge N, et al. Reliability and validity of two hand dynamometers when used by community-dwelling adults aged over 50 years. BMC Geriatr. 2022;22(1):580. 10.1186/s12877-022-03270-6 35840905 10.1186/s12877-022-03270-6 PMC9284760

[86] Benton MJ, Spicher JM, Silva-Smith AL. Validity and reliability of handgrip dynamometry in older adults: a comparison of two widely used dynamometers. PLoS ONE. 2022;17(6):e0270132. 10.1371/journal.pone.0270132 35727792 10.1371/journal.pone.0270132 PMC9212147

[87] Leong DP, Teo KK, Rangarajan S, Lopez-Jaramillo P, Avezum A Jr., Orlandini A, et al. Prognostic value of grip strength: findings from the prospective Urban Rural Epidemiology (PURE) study. Lancet. 2015;386(9990):266–73. 10.1016/S0140-6736(14)62000-6 25982160 10.1016/S0140-6736(14)62000-6

[88] Kubicki A. Functional assessment in older adults: should we use timed up and go or gait speed test? Neurosci Lett. 2014;577:89–94. 10.1016/j.neulet.2014.06.014 24933540 10.1016/j.neulet.2014.06.014

[89] Donoghue OA, Savva GM, Cronin H, Kenny RA, Horgan NF. Using timed up and go and usual gait speed to predict incident disability in daily activities among community-dwelling adults aged 65 and older. Arch Phys Med Rehabil. 2014;95(10):1954–61. 10.1016/j.apmr.2014.06.008 24977931 10.1016/j.apmr.2014.06.008

[90] Odegaard AO, Pereira MA, Koh WP, Arakawa K, Lee HP, Yu MC. Coffee, tea, and incident type 2 diabetes: the Singapore Chinese Health Study. Am J Clin Nutr. 2008;88(4):979–85. 10.1093/ajcn/88.4.979 18842784 10.1093/ajcn/88.4.979 PMC2737528

[91] Odegaard AO, Koh WP, Arakawa K, Yu MC, Pereira MA. Soft drink and juice consumption and risk of physician-diagnosed incident type 2 diabetes: the Singapore Chinese Health Study. Am J Epidemiol. 2010;171(6):701–8. 10.1093/aje/kwp452 20160170 10.1093/aje/kwp452 PMC2842218

