# Supplementary material for: Relationship between handgrip strength and timed up-and-go test on hospitalization costs in older adults: a population-based study
Source: BMC Public Health. 2025 Jan 23;25:290. doi: 10.1186/s12889-025-21489-x (PMC11760691; doi:10.1186/s12889-025-21489-x)
Supplement: Supplementary file 1 — Additional File 1: Supplementary Tables S1 to S3 [file 12889_2025_21489_MOESM1_ESM.docx]

**Relationship between handgrip strength and timed up-and-go test on hospitalization costs in older adults: a population-based study**

**Supplementary Material**

**Table of Contents**

**Supplementary Table S1:** Evaluation of collinearity among the covariates included in the statistical models.**2**

**Supplementary Table S2:** Marginal effects of handgrip strength and TUG time on the mean increase in hospitalization costs during the subsequent year, when the physical tests were modeled continuously and participants with outlying values were excluded from analysis.**3**

**Supplementary Table S3:** Marginal effects of self-reported history of physician-diagnosed comorbidities on the mean increase in hospitalization costs during the subsequent year.**4**

**Supplementary Table S1:** Evaluation of collinearity among the covariates included in the statistical models.

| **Covariate** | **Generalized variance inflation factor (GVIF)** |
| --- | --- |
| Age | 1.43 |
| Sex | 1.78 |
| Level of education | 1.41 |
| Housing type | 1.20 |
| Living arrangement | 1.38 |
| Smoking status | 1.48 |
| BMI group | 1.94 |
| Waist circumference | 1.96 |
| Hypertension | 1.27 |
| Coronary artery disease | 1.08 |
| Stroke | 1.03 |
| Diabetes | 1.11 |
| Hypercholesterolaemia | 1.24 |
| Gout | 1.05 |
| Cancer | 1.01 |
| Chronic lung disease | 1.01 |
| Bone fractures | 1.01 |
| Arthritis | 1.09 |
| Kidney failure | 1.02 |
| Parkinson’s disease | 1.01 |
| Cognitive impairment | 1.08 |
| Depression | 1.40 |
| Anxiety | 1.34 |
| Functional disability | 1.23 |

**Supplementary Table S2:** Marginal effects of handgrip strength and TUG time on the mean increase in hospitalization costs during the subsequent year, when the physical tests were modeled continuously and participants with outlying values were excluded from analysis.

|  | **Mean increase in hospitalization costs (95% CI) [2016 USD]** | | | |
| --- | --- | --- | --- | --- |
|  | **Model 1 ^a^** | **Model 2 ^b^** | **Model 3 ^c^** | **Model 4 ^d^** |
| **Per 5 kg decrease in handgrip strength** | $456 ($346, $566) | $486 ($371, $601) | $349 ($230, $467) | $242 ($121, $363) |
| **Per SD (2.9 sec) increase in TUG time** | $483 ($376, $590) | $491 ($375, $607) | $386 ($268, $505) | $332 ($211, $454) |

Presented as 2016 United States dollars (USD) [95% confidence interval (CI)]. CI: confidence interval; BMI: body mass index; TUG: timed up-and-go.

^a^ Model 1: adjusted for age, sex.

^b^ Model 2: adjusted for Model 1 and level of education, housing type, living arrangement, smoking status, BMI group, waist circumference.

^c^ Model 3: adjusted for Model 2 and hypertension, coronary artery disease, stroke, diabetes, hypercholesterolaemia, gout, cancer, chronic lung disease, bone fractures, arthritis, kidney failure, Parkinson’s disease, cognitive impairment, depression, anxiety, functional disability.

^d^ Model 4: adjusted for Model 3 and TUG test time or handgrip strength.

**Supplementary Table S3:** Marginal effects of self-reported history of physician-diagnosed comorbidities on the mean increase in hospitalization costs during the subsequent year.

| **Self-reported history of comorbidity** | **Mean increase in hospitalization costs (95% CI) [2016 USD] ^a^** |
| --- | --- |
| Hypertension | $319 ($88, $549) |
| Coronary artery disease | $1006 ($577, $1435) |
| Stroke | -$54 (-$480, $371) |
| Diabetes | $396 ($125, $667) |
| Hypercholesterolaemia | -$198 (-$439, $43) |
| Gout | $172 (-$304, $649) |
| Cancer | $845 ($365, $1324) |
| Chronic lung disease | $718 (-$259, $1694) |
| Bone fractures | $416 ($13, $819) |
| Arthritis | $177 (-$95, $450) |
| Kidney failure | $3106 ($1440, $4772) |
| Parkinson’s disease | $649 (-$1042, $2339) |

Presented as 2016 United States dollars (USD) [95% confidence interval (CI)]. CI: confidence interval; BMI: body mass index; TUG: timed up-and-go.

^a^ Adjusted for age, sex, level of education, housing type, living arrangement, smoking status, BMI group, waist circumference, cognitive impairment, depression, anxiety, functional disability, handgrip strength, TUG test time.
